# Myeloid-Related Protein-14 Contributes to Protective Immunity in Gram-Negative Pneumonia Derived Sepsis

**DOI:** 10.1371/journal.ppat.1002987

**Published:** 2012-10-25

**Authors:** Ahmed Achouiti, Thomas Vogl, Constantin F. Urban, Marc Röhm, Tijmen J. Hommes, Marieke A. D. van Zoelen, Sandrine Florquin, Johannes Roth, Cornelis van 't Veer, Alex F. de Vos, Tom van der Poll

**Affiliations:** 1 Center for Experimental and Molecular Medicine, Academic Medical Center, University of Amsterdam, Amsterdam, The Netherlands; 2 Center for Infection and Immunity, Academic Medical Center, University of Amsterdam, Amsterdam, The Netherlands; 3 Institute of Immunology, University of Münster, Münster, Germany; 4 Clinical Microbiology Department, Laboratory for Molecular Infection Medicine Sweden, Umeå University, Umeå, Sweden; 5 Department of Pathology, Academic Medical Center, University of Amsterdam, Amsterdam, The Netherlands; 6 Division of Infectious Diseases, Academic Medical Center, University of Amsterdam, Amsterdam, The Netherlands; Max-Planck Institute for Infection Biology, Germany

## Abstract

*Klebsiella (K.) pneumoniae* is a common cause of pneumonia-derived sepsis. Myeloid related protein 8 (MRP8, S100A8) and MRP14 (S100A9) are the most abundant cytoplasmic proteins in neutrophils. They can form MRP8/14 heterodimers that are released upon cell stress stimuli. MRP8/14 reportedly exerts antimicrobial activity, but in acute fulminant sepsis models MRP8/14 has been found to contribute to organ damage and death. We here determined the role of MRP8/14 in *K. pneumoniae* sepsis originating from the lungs, using an established model characterized by gradual growth of bacteria with subsequent dissemination. Infection resulted in gradually increasing MRP8/14 levels in lungs and plasma. *Mrp14* deficient *(mrp14^−/−^)* mice, unable to form MRP8/14 heterodimers, showed enhanced bacterial dissemination accompanied by increased organ damage and a reduced survival. *Mrp14^−/−^* macrophages were reduced in their capacity to phagocytose *Klebsiella*. In addition, recombinant MRP8/14 heterodimers, but not MRP8 or MRP14 alone, prevented growth of *Klebsiella in vitro* through chelation of divalent cations. Neutrophil extracellular traps (NETs) prepared from wildtype but not from *mrp14^−/−^* neutrophils inhibited *Klebsiella* growth; in accordance, the capacity of human NETs to kill *Klebsiella* was strongly impaired by an anti-MRP14 antibody or the addition of zinc. These results identify MRP8/14 as key player in protective innate immunity during *Klebsiella* pneumonia.

## Author Summary

Neutrophils are phagocytes that are well known for their capacity to engulf and kill microbial pathogens. It has become increasingly clear that neutrophils also kill or inhibit growth extracellularly by releasing neutrophil extracellular traps (NETs), chromatin fibers decorated with neutrophil derived proteins. MRP8/14 has been identified as one of the major antimicrobial proteins herein. Previous investigations have shown that endogenously released MRP8/14 is also sensed by the host as a danger signal and able to potentiate the harmful systemic inflammatory response syndrome. Indeed, in the setting of fulminant systemic inflammation, such as induced by endotoxin or *Escherichia coli* administration, MRP8/14 contributed to organ injury and mortality. The clinical scenario of sepsis however, involves an initial infection at the primary site followed by bacterial spreading to other organs. In the present setting of pneumonia-derived sepsis using the common human respiratory and sepsis pathogen *Klebsiella pneumoniae* MRP8/14 clearly served a beneficial role in antimicrobial defense. We here provide a likely mechanism by showing that MRP8/14 plays a role in phagocytosis and that its presence is critical in both murine and human NETs to inhibit bacterial growth.

## Introduction

*Klebsiella (K.) pneumoniae* is a frequent causative pathogen in pneumonia [1], [2] and the second most common cause of gram-negative sepsis [3], [4]. *Klebsiella* infection presents a significant burden on healthcare and is associated with high morbidity and mortality rates. Effective treatment of this microorganism is even more challenging due to the emergence of microbial resistance to (last-resort) antibiotics [5], [6]. It is therefore of great importance to expand our understanding on host defense mechanisms that influence the outcome of *Klebsiella* pneumonia. Such knowledge may eventually help in the development of new therapies.

Invasive infection and accompanying inflammatory mechanisms can cause tissue damage that is associated with release of endogenous “alarm” proteins. These proteins, also known as Damage Associated Molecular Patterns (DAMPs), are recognized by pattern recognition receptors and perpetuate inflammatory responses [7], [8]. Among these DAMPs, the S100 proteins MRP8 (myeloid-related protein, S100A8) and MRP14 (S100A9) have gained increasing interest [9], [10]. They are mainly and constitutively expressed in neutrophils where they comprise 45 percent of total cytoplasmic protein [11]. MRP8 and MRP14 are able to dimerize with a clear preference for the most stable and biologically relevant MRP8/14 heterodimer (or calprotectin), which can be actively released into the extracellular space [12]–[15]. MRP8/14 induces a variety of host responses and the extent of expression correlates with clinical [9], [10] and experimental [16] disease activity. Previous investigations have pointed to a complex role of MRP8/14 in severe infection, which may either be protective or harmful to the host. MRP8/14 can enhance inflammation via activation of Toll-like receptor (TLR)4, by amplifying tumor necrosis factor (TNF)-α release in response to lipopolysaccharide (LPS), the immunostimulatory component of the gram-negative bacterial cell wall. In the setting of fulminant systemic inflammation such as induced by high dose LPS or *Escherichia (E.) coli* administration, endogenous MRP8/14 contributes to lethality [15]. On the opposite site, MRP8/14 may be important for innate defense against microorganisms by virtue of its involvement in leukocyte migration [17]–[20] and its direct antimicrobial effects [21]–[23]. In addition, recent studies revealed that MRP8/14 is a major component of neutrophil extracellular traps (NETs) [24], DNA-networks released by neutrophils that trap microorganisms and facilitate interaction with antimicrobial proteins and thereby bacterial killing [25], [26]. Although at present the importance for NET associated MRP8/14 for bacterial killing is unknown, the presence of MRP8/14 was found to be crucial for the clearance of fungi by NETs *in vitro* [24], [27].

In the present study, we aimed to characterize the role of MRP8/14 during pneumonia-originating sepsis caused by *K. pneumoniae*. For this we used *mrp14* deficient (*mrp14^−/−^*) mice, which due to instability of MRP8 in the absence of its binding partner MRP14 are considered deficient for MRP8/14 at protein level [28]–[30]. We here show that MRP8/14 deficiency results in enhanced bacterial dissemination, increased distant organ damage and a reduced survival during *Klebsiella* pneumonia. Using *in vitro* models, we show that *mrp14^−/−^* macrophages have a reduced capacity to phagocytose this bacterium. We further demonstrate that MRP8/14 directly reduces the growth of *Klebsiella* and in addition is essential in NET-mediated growth inhibition of this pathogen. These results identify MRP8/14 as an important protective mediator in the innate immune response to bacterial pneumonia caused by a clinically relevant pathogen.

## Results

### MRP8/14 levels increase during pneumonia

**Figure 1:**
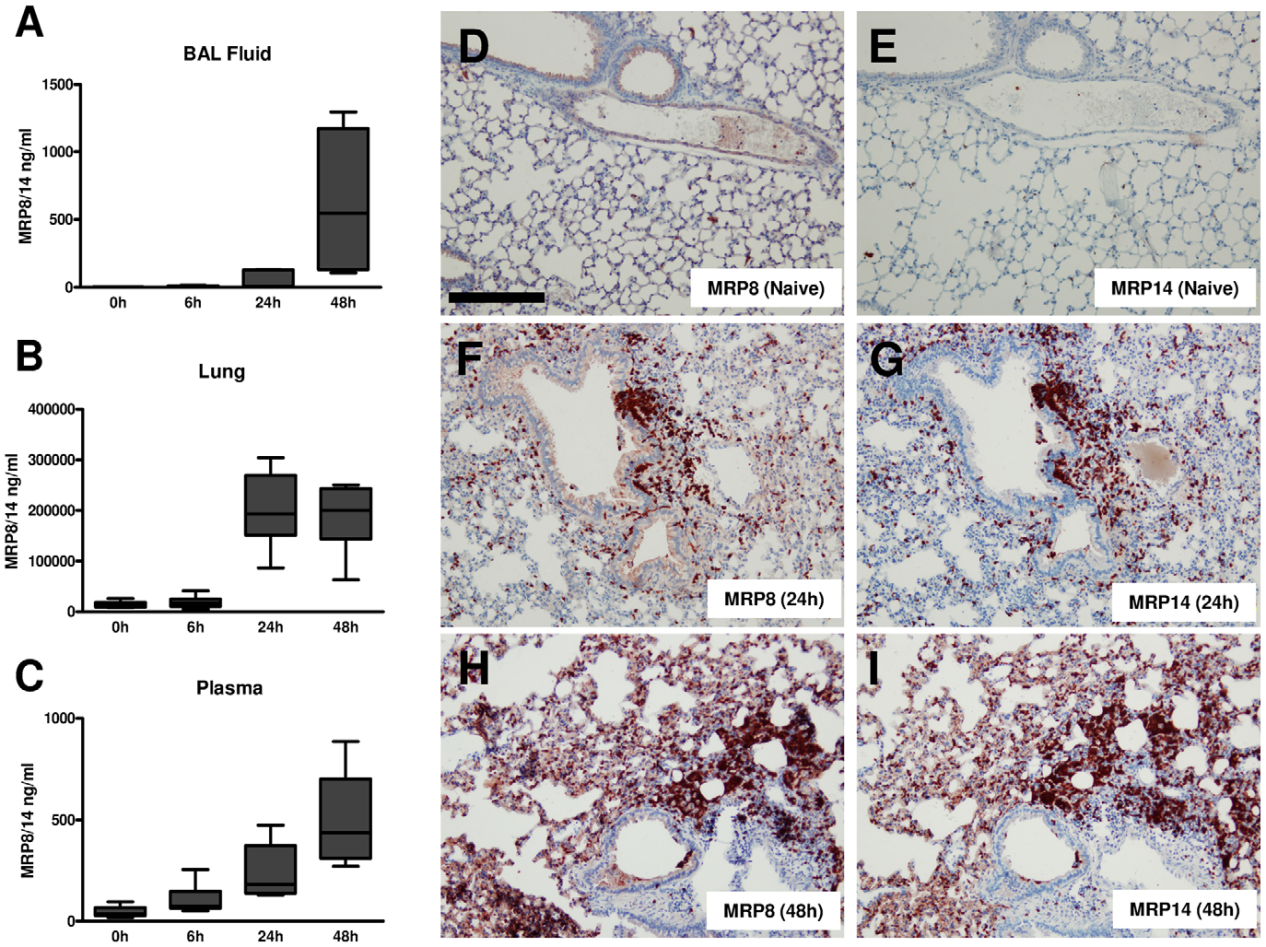
*K. pneumoniae* pneumonia results in an increase of endogenous MRP8/14 levels and enhanced MRP8 and MRP14 expression in lungs. MRP8/14 levels in BAL fluid (A), whole lung homogenates (B), and plasma (C) were determined in naive mice (n = 4) and 6, 24 and 48 hours after intranasal *K. pneumoniae* infection (n = 6–8). Data are expressed as box-and-whisker diagrams depicting the smallest observation, lower quartile, median, upper quartile and largest observation. MRP8 and MRP14 were stained in lungs of naive and infected mice. Shown here are representative slides of MRP8 (D) and MRP14 (E) staining of naive mice, MRP8 (F) and MRP14 (G) staining 24 hours and MRP8 (H) and MRP14 (I) staining 48 hours after infection. Scalebar indicates 200 µm.

To gain a first insight into the potential role of MRP8/14 in gram-negative pneumonia, we intranasally infected Wt mice with *K. pneumoniae* (10^4^ cfu) and measured local and systemic MRP8/14 concentrations 6, 24 and 48 hours thereafter. MRP8/14 levels became detectable in BAL fluid at 24 hours after infection; high levels were found at 48 hours (median 745 ng/ml; Fig. 1A). In whole lung homogenates, MRP8/14 was detectable at low levels in uninfected mice and concentrations did not increase during the first 6 hours after infection; high MRP8/14 levels were detected at later time points (median 200 µg/ml at 24 hours; Fig. 1B). Plasma MRP8/14 levels also increased during the course of the infection, reaching peak concentrations at 48 hours (median 440 ng/ml; Fig. 1C).

To obtain insight into the cellular source of MRP8/14, we stained lung tissue slides obtained from naïve and infected Wt mice for MRP8 and MRP14. Bronchial epithelium of naïve lungs showed a faint staining for MRP8 (but not MRP14) that did not intensify during *Klebsiella* pneumonia. A small number of MRP8 and MRP14 positive cells, mainly residing macrophages, were already present. During the course of the disease, expression of both MRP8 and MRP14 increased strongly, primarily as a consequence of infiltrating neutrophils (Fig. 1D–I).

### Mrp14 deficiency impairs host defense during pneumonia

**Figure 2:**
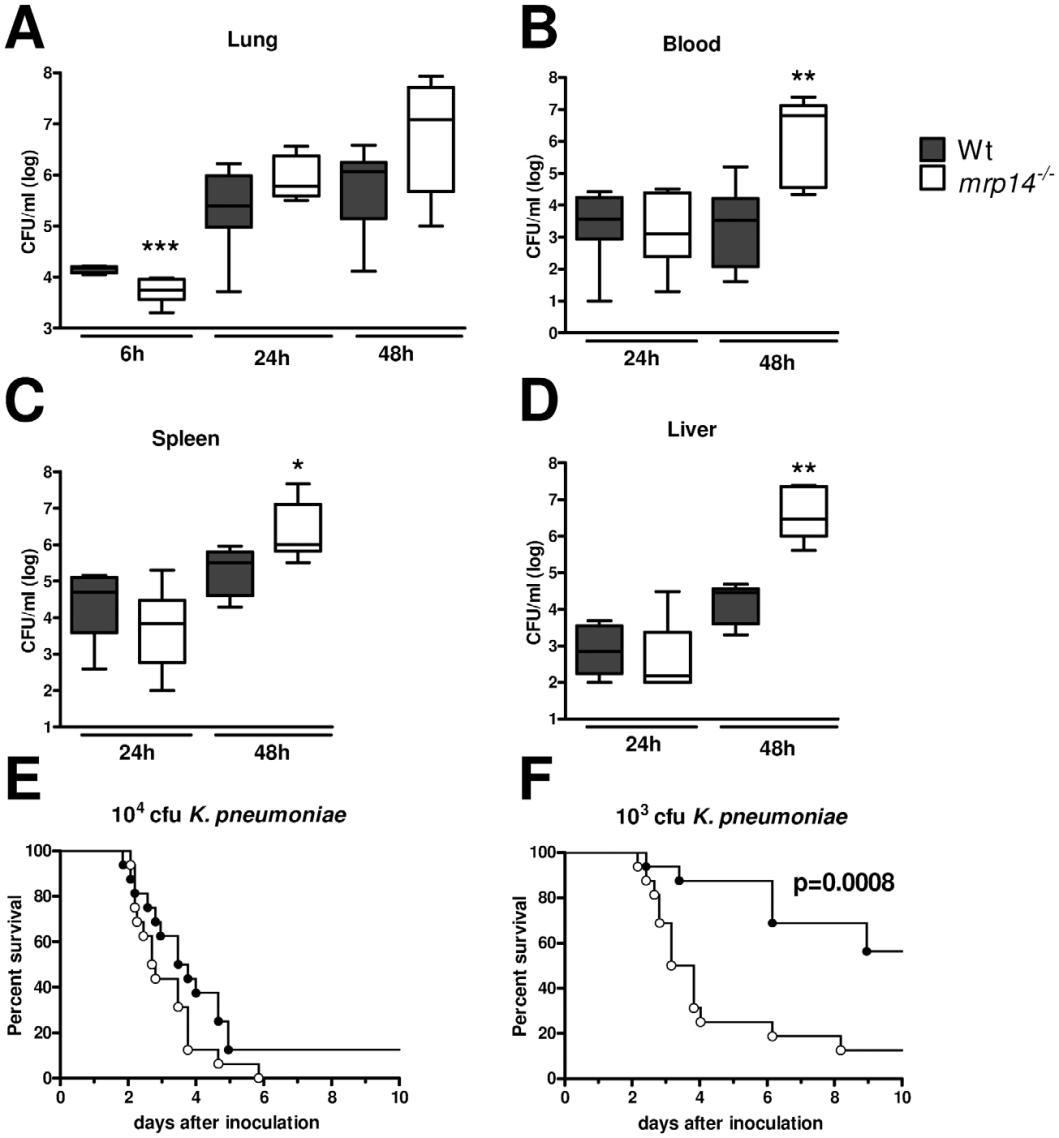
*Mrp14^−/−^* mice show enhanced bacterial dissemination and increased mortality during pneumonia derived sepsis caused by *K. pneumoniae*. Bacterial loads in the lung (A), blood (B), spleen (C) and liver (D) of *K. pneumoniae* in Wt (grey) and *mrp14^−/−^* mice (white). Data are expressed as box-and-whisker diagrams depicting the smallest observation, lower quartile, median, upper quartile and largest observation (8 mice per group at each time point). * p<0.05, ** p<0.01 versus Wt mice at the same time point. Survival of Wt and *mrp14^−/−^* mice after intranasal inoculation of 10.000 (E) or 1.000 cfu (F) (n = 12–16 per group in each experiment).

To investigate the functional role of MRP8/14 in host defense during gram-negative pneumonia, we infected *mrp14^−/−^* and Wt mice with 10^4^ viable *K. pneumoniae* and harvested lungs, blood, spleen and livers at predefined time points for quantitative cultures, seeking to collect data representative for local defense, at the primary site of infection, and subsequent dissemination. Initially, *mrp14^−/−^* mice showed slightly lower bacterial loads in lungs compared to Wt mice (Fig. 2A; p<0.001). At later time points, *mrp14^−/−^* mice tended to have higher bacterial burdens in their lungs. Remarkably, at late stage infection (after 48 hours, shortly before the first deaths occurred) *mrp14^−/−^* mice displayed increased bacterial loads in blood, liver (both p<0.01) and spleen (p<0.05), suggesting that MRP14 deficiency is associated with enhanced dissemination of the infection (Fig. 2B–D). To investigate the impact of the reduced antibacterial defense in *mrp14^−/−^* mice on survival, we performed an observational study instilling the same infectious dose (10^4^ cfu) as used in the experiments determining bacterial growth and spreading (Fig. 2E). This inoculum rapidly led to death shortly after 48 hours in all mice; notably, *mrp14^−/−^* mice tended to die earlier in this lethal model (p = 0.10 versus Wt mice). Arguing that the infectious challenge might have been too high to reveal a detrimental effect of MRP14 deficiency on survival, we repeated this observational study with a 10-fold lower inoculum (10^3^ cfu; Fig. 2F). This infectious dose resulted in a 56% lethality in Wt mice 10 days after inoculation; *mrp14^−/−^* mice displayed a higher mortality rate and eventually 94% of *mrp14^−/−^* mice died (p = 0.0008 versus Wt mice). These data establish the important protective role of MRP8/14 in *K. pneumoniae* pneumonia as reflected by an increased dissemination of bacteria and a reduced survival.

The fact that MRP14 deficiency in particular influenced bacterial loads in distant organs led us to hypothesize that *mrp14^−/−^* mice would also show enhanced bacterial growth after direct intravenous injection of *K. pneumoniae*. Indeed, 48 hours after intravenous infection with 2×10^3^ *Klebsiella* cfu, *mrp14^−/−^* mice demonstrated higher bacterial burdens in spleen, liver and lungs (all p<0.01; Fig. S1). These data suggest that MRP8/14 importantly contributes to systemic protection against *K. pneumoniae* infection.

### MRP14 deficiency does not influence neutrophil recruitment into the lungs

**Figure 3:**
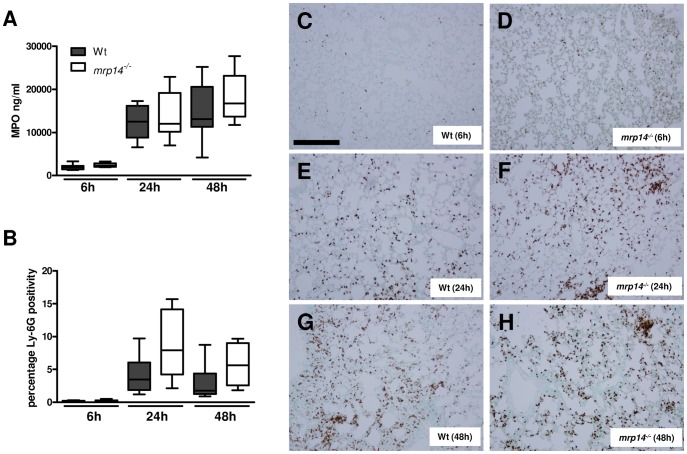
Neutrophil influx in *Klebsiella* pneumonia is not influenced by MRP14 deficiency. MPO levels in whole lung homogenates (A) and quantitation of pulmonary Ly-6G positivity 6, 24 and 48 hours after infection (B) in Wt (grey) and *mrp14^−/−^* mice (white). Data are expressed as box-and-whisker diagrams depicting the smallest observation, lower quartile, median, upper quartile and largest observation (8 mice per group at each time point). There were no statistically significant differences between the groups. Representative neutrophil stainings (brown) of Wt (C) and *mrp14^−/−^* mice (D) 6 hours, Wt (E) and *mrp14^−/−^* mice (F) 24 hours and Wt (G) and *mrp14^−/−^* mice (H) 48 hours after induction of *K. pneumoniae* pneumonia.. Scalebar indicates 200 µm.

Bacterial pneumonia is associated with neutrophil migration to the lung parenchyma, which is considered to be an essential component of a protective innate immune response [31], [32]. Previous studies indeed have documented that neutrophils play an important role in innate defense early after *Klebsiella* airway infection [33], [34]. MRP8/14 has been implicated as an important mediator of neutrophil recruitment in various inflammatory conditions [17]–[19], [35], including pneumonia [20]. Therefore, we determined the extent of neutrophil influx in *mrp14^−/−^* and Wt mice at 6, 24 and 48 hours after intranasal challenge with *K. pneumoniae* by assessing the number of Ly-6G positive cells in lung tissue sections and by measuring MPO concentrations in whole lung homogenates (Fig. 3). Neither the number of Ly-6G positive cells, nor whole lung MPO concentrations differed between *mrp14^−/−^* and Wt mice at any time point; if anything, *mrp14^−/−^* mice tended to have more Ly-6G positive cells and higher MPO levels in their lungs than Wt mice. Hence, these data strongly argue against a role for MRP8/14 in neutrophil influx into the lungs during *Klebsiella* pneumonia.

### MRP14 deficiency results in increased lung pathology

**Figure 4:**
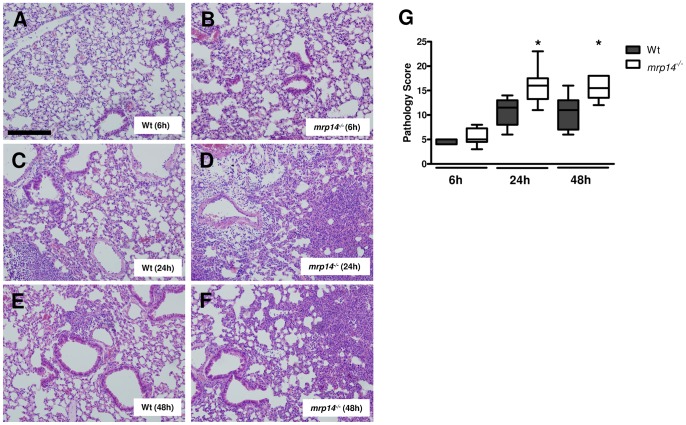
*Mrp14^−/−^* mice show enhanced lung pathology during *K. pneumoniae* pneumonia. Representative slides of lung haematoxylin and eosin (HE) staining of Wt (A) and *mrp14^−/−^* mice (B) 6 hours, Wt (C) and *mrp14^−/−^* mice (D) 24 hours and Wt (E) and *mrp14^−/−^* mice (F) 48 hours after intranasal *K. pneumoniae* infection. Scalebar indicates 200 µm. Total pathology score at indicated time points post infection in Wt (grey) and *mrp14^−/−^* mice (white) was determined according to the scoring system described in the Methods section (G). Data are expressed as box-and-whisker diagrams depicting the smallest observation, lower quartile, median, upper quartile and largest observation (8 mice per group at each time point). * p<0.05 versus Wt mice at the same time point.

This model of *K. pneumoniae* pneumonia is associated with profound lung inflammation [36]–[38]. Although the presence of MRP8/14 did not affect lung bacterial loads or neutrophil recruitment at later time points, we wondered whether lung pathology would be influenced by the (proinflammatory) effects of MRP8/14 itself during *Klebsiella* pneumosepsis. We therefore analyzed HE-stained lung tissue slides obtained from infected Wt and *mrp14^−/−^* mice using the semi-quantitative scoring system described in the Materials en Methods section (Fig. 4). Already at 6 hours after infection mild interstitial inflammation and pleuritis were found in all mice; at later stages endothelialitis, bronchitis and edema became apparent. Interestingly, after 24 and 48 hours, *mrp14^−/−^* mice showed exaggerated lung pathology with enhanced interstitial inflammation, bronchitis and larger surfaces of confluent inflammation infiltrate. The enhanced lung pathology may be a reflection of the increased disease severity in *mrp14* ^−/−^ mice and suggest that the presence of MRP8/14, though proinflammatory, is not essential for the induction of lung inflammation during *Klebsiella* pneumosepsis.

### Impact of MRP14 deficiency on cytokine and chemokine levels

**Figure 5:**
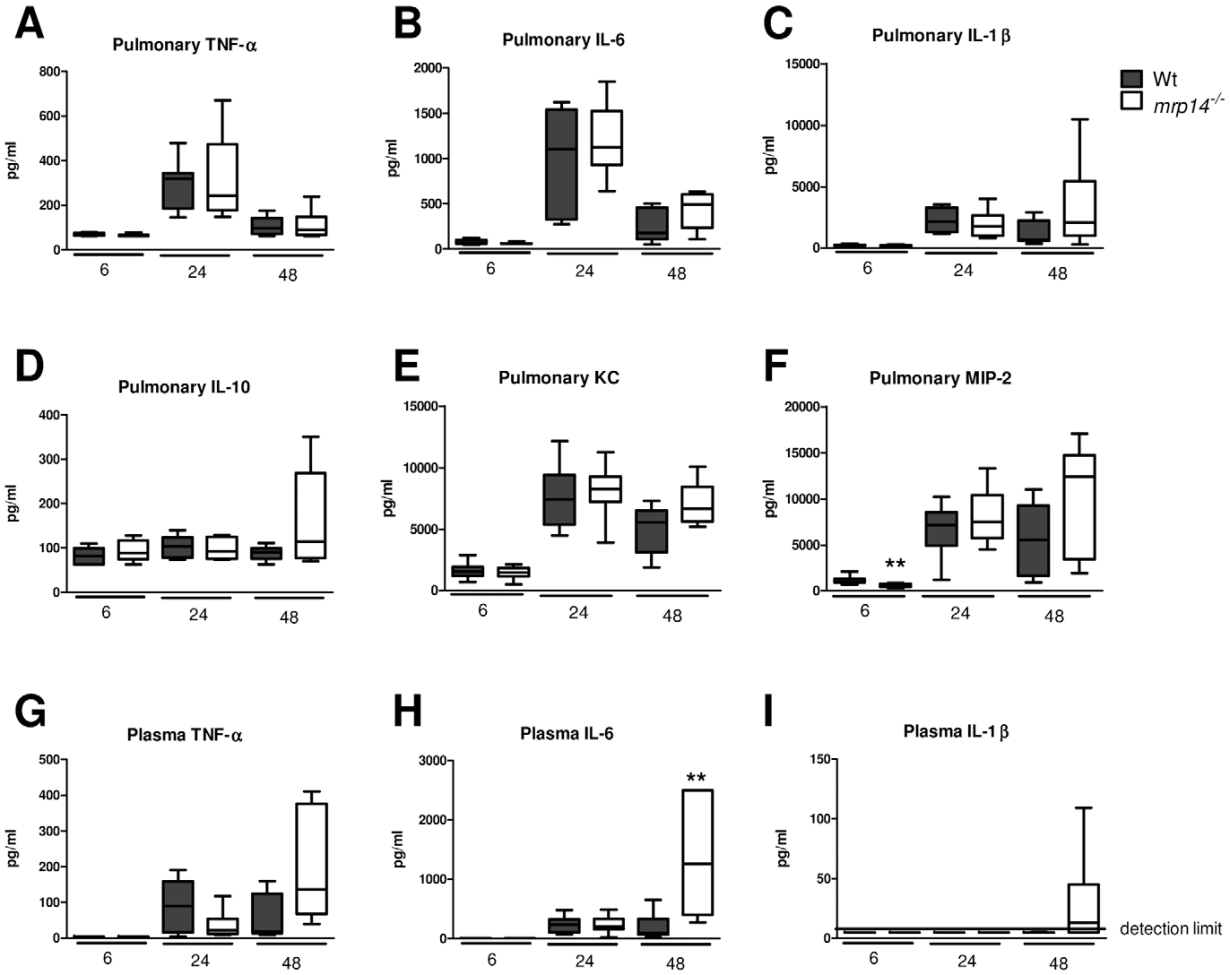
Cytokine and chemokine levels in lungs and plasma. Lung cytokine (TNF-α, L-1β, IL-6, IL-10) (A–D), chemokine (KC and MIP-2) (E–F) and plasma cytokine (TNF-α, L-1β, IL-6 ) levels (G–I), 6, 24 and 48 hours after intranasal *K. pneumoniae* infection in Wt (grey) and *mrp14^−/−^* mice (white). Data are expressed as box-and-whisker diagrams depicting the smallest observation, lower quartile, median, upper quartile and largest observation (8 mice per group at each time point). * p<0.05, ** p<0.01 versus Wt mice.

Extracellular MRP8/14 has been shown to amplify the TNF-α response upon LPS stimulation and *mrp14^−/−^* bone marrow cells demonstrated a reduced responsiveness to LPS *in vitro* [15]. *In vivo* this correlated with lower plasma TNF-α levels in *mrp14^−/−^* mice challenged with LPS [15]. To study the impact of MRP14 deficiency on cytokine release in gram-negative pneumonia, we measured the levels of cytokines (TNF-α, IL-1β, IL-6, IL-10) and chemokines (MIP-2, KC) in lung homogenates and plasma (TNF-α, IL-1β and IL-6 only) harvested from *mrp14^−/−^* and Wt mice after intranasal infection with *Klebsiella*. Surprisingly, overall differences between mouse strains were limited. In whole lung homogenates, *mrp14^−/−^* mice displayed reduced MIP-2 levels after 6 hours only; at other time points and for other mediators, levels were similar between groups (Fig. 5A–F). Similarly, plasma cytokine levels did not differ between Wt and *mrp14^−/−^* mice at 6 or 24 hours post infection; after 48 hours however the plasma levels of IL-6 and IL-1β were even higher in *mrp14^−/−^* mice compared to the Wt mice (Fig. 5G–I).

We next determined the role of endogenous MRP8/14 in the inflammatory response to *Klebsiella* infection *in vitro*. To this end, we measured TNF-α levels after incubating whole blood from Wt, *mrp14^−/−^* and *tlr4^−/−^* mice with log-increasing loads of viable, growth-arrested *Klebsiella* for 6 hours. In consistence with our *in vivo* data, whole blood obtained from Wt and *mrp14^−/−^* mice displayed a similar cytokine response. Only upon exposure to the highest bacterial concentration, *mrp14^−/−^* whole blood showed a modestly reduced cytokine response. Whole blood from *tlr4^−/−^* mice showed almost no TNF-α release in response to *Klebsiella* (Fig. S2). Together, these data indicate that endogenous MRP8/14 has no, or little contribution to the TLR4 mediated cytokine response to *K. pneumoniae* infection.

### *Mrp14^−/−^* mice demonstrate enhanced liver damage

**Figure 6:**
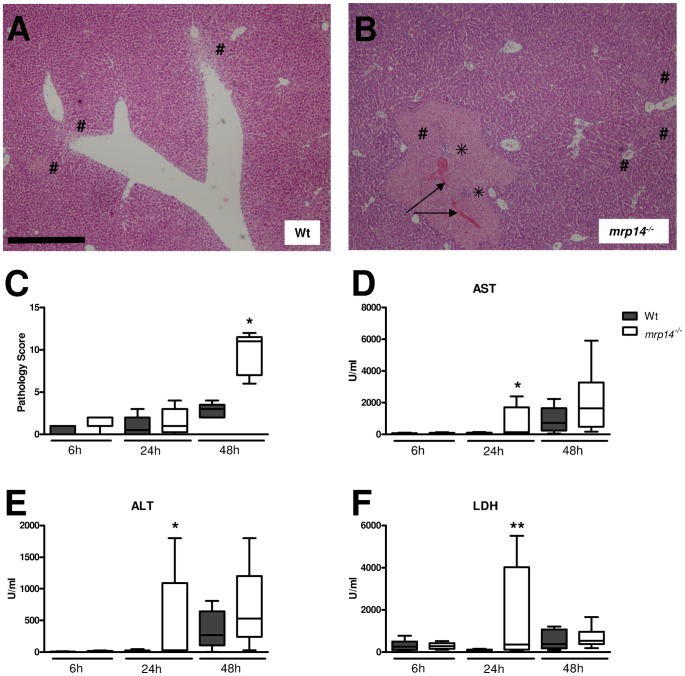
*Mrp14^−/−^* mice show enhanced hepatocellular injury during pneumonia derived sepsis caused by *Klebsiella*. Representative slides of liver haematoxylin and eosin HE staining of Wt (A) and *mrp14^−/−^* mice (B) 48 hours post infection. Livers from *mrp14^−/−^* mice displayed more advanced liver necrosis (#) accompanied with (micro) abscesses (<$>\vskip -1pt\scale 70%\raster="rg1"<$>). Arrows indicate fibrin deposits as a sign of thrombosis. Scalebar indicates 0.5 mm. Total pathology score was determined at indicated time points in Wt (grey) and *mrp14^−/−^* mice (white) according to the scoring system described in the Methods section (C). Aspartate aminotransferase (AST) (D), alanine aminotransferase (ALT) (E), and lactate dehydrogenase (LDH) (F) were measured in plasma. Data are expressed as box-and-whisker diagrams depicting the smallest observation, lower quartile, median, upper quartile and largest observation (8 mice per group at each time point). *p<0.05, **p<0.01 versus Wt mice at the same time point.

This model of gram-negative pneumonia-derived sepsis is associated with hepatocellular injury during late stage infection [38]. In our time point experiments, MRP14 deficiency was associated with enhanced bacterial dissemination to distant organs, including the liver. We were thus interested to what extent this enhanced bacterial dissemination influenced hepatocellular injury in these animals. Microscopic examination revealed dramatically enhanced pathology in livers from *mrp14^−/−^* mice after 48 hours, reflected by more advanced liver necrosis accompanied with thrombi and more (micro)abscesses compared to Wt mice (Fig. 6A–C). Clinical chemistry findings confirmed the existence of more extensive hepatocellular injury, *i.e. mrp14^−/−^* mice had higher plasma levels of ALT and AST, in particular 24 hours after infection (Fig. 6D,E). At this time point, *mrp14^−/−^* mice also showed higher plasma LDH concentrations (indicative for cellular injury in general) relative to Wt mice (Fig. 6F).

### MRP14 deficiency reduces macrophage phagocytosis of *K. pneumoniae*

**Figure 7:**
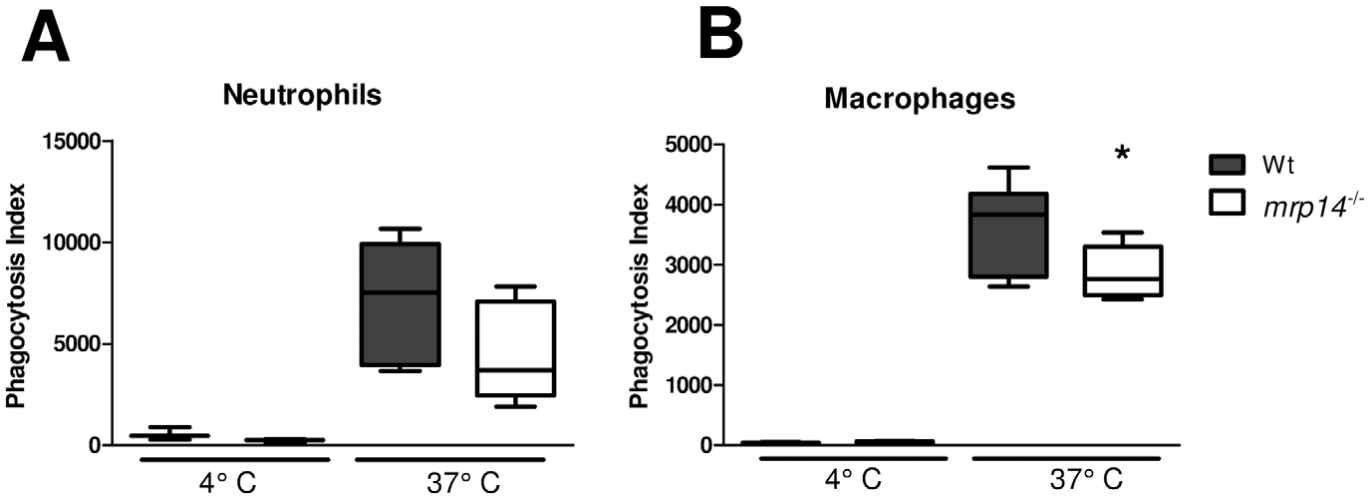
Phagocytosis is impaired in MRP14 deficient macrophages. Growth arrested, CFSE-labelled *K. pneumoniae* were incubated with peripheral blood neutrophils (A) or macrophages (B) from Wt (grey) and *mrp14^−/−^* mice (white) at 4°C (n = 3–4 per mouse strain) or 37°C (n = 6–8 per mouse strain) for 20 and 60 minutes respectively after which phagocytosis was quantified (see Materials and Methods). Data are expressed as box-and-whisker diagrams depicting the smallest observation, lower quartile, median, upper quartile and largest observation (8 mice per group at each time point). *p<0.05.

A recent report has shown that addition of MRP14 is able to enhance bactericidal effects of human neutrophils by means of improving bacterial phagocytosis capacity [39]. We wondered whether this would correspond to impaired *K. pneumoniae* phagocytosis in *mrp14^−/−^* murine phagocytes. To investigate this possibility, we harvested whole blood and macrophages from naïve Wt and *mrp14^−/−^* mice and compared neutrophil and macrophage ability to internalize CFSE-labelled, viable, growth-arrested *K. pneumoniae* by FACS. Although MRP8/14 is abundantly present in neutrophils, *mrp14^−/−^* neutrophils only displayed a modest reduction in their capacity to phagocytose *Klebsiella* compared to Wt neutrophils (Fig. 7A). *Mrp14^−/−^* macrophages however, were significantly reduced in their capacity to internalize *Klebsiella* bacteria (Fig. 7B). In addition to increased dissemination, the reduced capacity of macrophages to phagocytose the bacteria most probably contributed to the enhanced bacterial outgrowth in *mrp14^−/−^* mice during *Klebsiella* infection *in vivo*.

### MRP8/14 inhibits growth of *Klebsiella in vitro* through metal chelation

**Figure 8:**
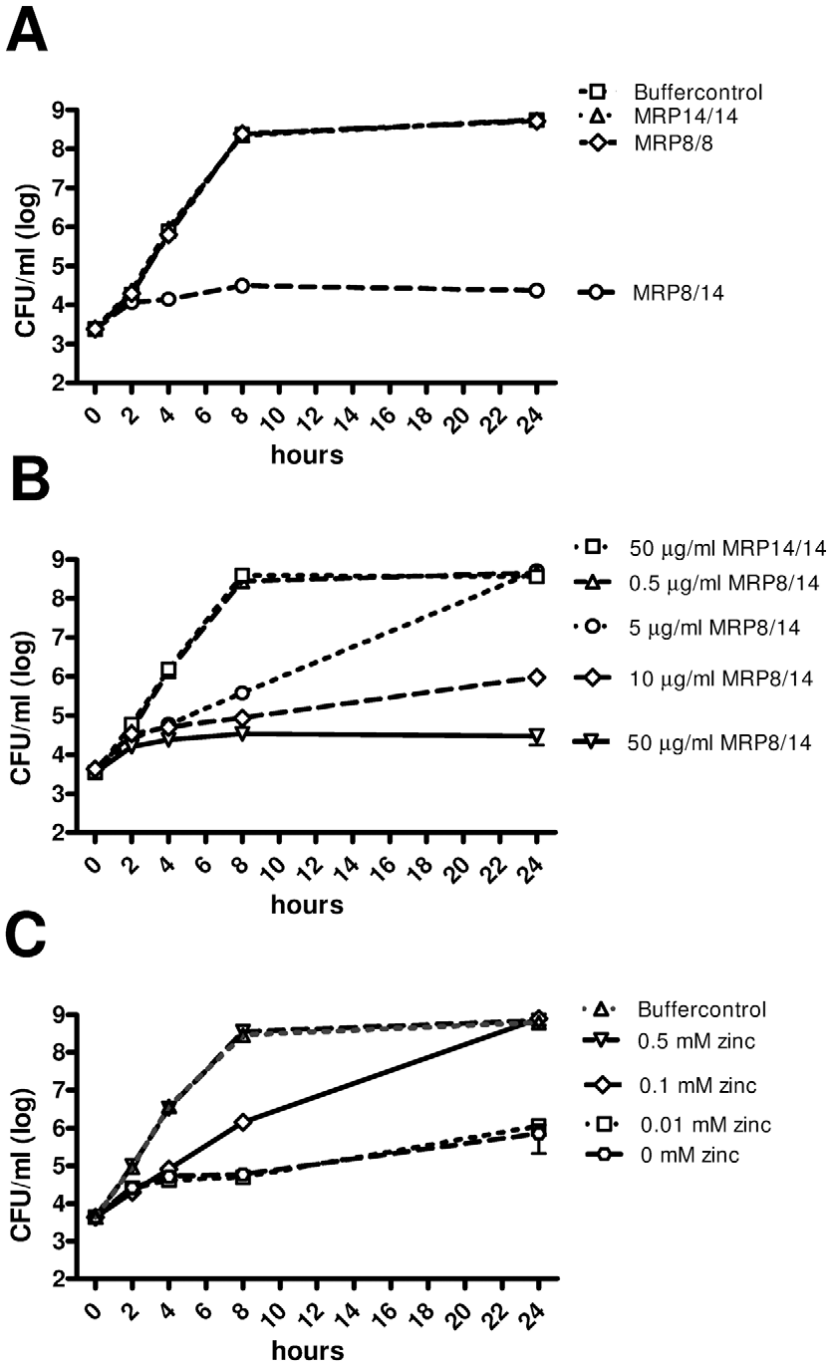
MRP8/14 reduces bacterial growth of *K. pneumoniae* through metal chelation. Growth of *K. pneumoniae* was assessed for a maximum of 24 hours in the presence of recombinant MRP8/14, MRP8 homodimer or MRP14 homodimer (50 µg/ml) (A). Bacterial growth was dose dependently inhibited by MRP8/14 (B); the growth inhibiting effect of MRP8/14 (10 µg/ml) was reversed by the addition of zinc (C). Data are means ± SEs of at least 3 replicates and representative of triplicate experiments.

MRP8/14 has been shown to inhibit the growth of several microorganisms by binding of divalent cations [22], [24], [27]. To study whether *K. pneumoniae* growth is affected by MRPs, we grew bacteria in medium for up to 24 hours in the presence or absence of MRP8/14 heterodimer, MRP8 homodimer or MRP14 homodimer (all 50 µg/ml). Addition of MRP8/14 heterodimer almost abolished growth of *K. pneumoniae*, while MRP8 and MRP14 homodimers had no effect (Fig. 8A). The growth inhibitory effect of MRP8/14 was dose dependent (Fig. 8B). To check whether MRP8/14 induced growth inhibition was due to a metal chelating effect, zinc was added to medium treated with 10 µg/ml MRP8/14 prior to incubation with bacteria; in this experiment (Fig. 8C) growth was restored when zinc was added in increasing amounts. Thus, MRP8/14 inhibits growth of *K. pneumoniae* through chelation of metals.

### Growth inhibition of *Klebsiella* by mouse and human NETs is MRP8/14 dependent

**Figure 9:**
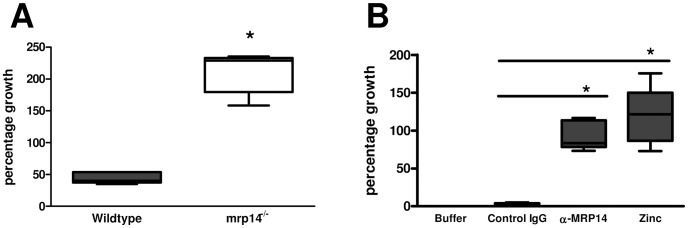
Growth inhibition of *Klebsiella* by mouse and human NETs is MRP8/14 dependent. 3×10^5^ mouse neutrophils isolated from Wt and *mrp14^−/−^* mice were induced to make neutrophil extracellular traps (NETs) and subsequently infected with 5000 cfu *K. pneumoniae*. Cfu counts were determined after incubation of 7 hours at 37°C (A). 5×10^5^ human neutrophils were induced to make NETs and pretreated with a rabbit polyclonal anti-MRP14 antibody (α-MRP14), an unspecific rabbit polyclonal control antibody (control IgG) or an excess of zinc and then infected with 100 cfu *Klebsiella*. Cfu counts were determined after incubation of 10 hours (B). Percentage bacterial growth in the presence of NETs was calculated based on bacterial counts relative to medium controls without NETs. Data are expressed as box-and-whisker diagrams depicting the smallest observation, lower quartile, median, upper quartile and largest observation of at least 5 replicates. *p<0.05 versus controls.

Earlier studies have demonstrated that *Klebsiella pneumoniae* induces NET formation *in vivo* [40]. Using immunofluorescence technique, we found similar decondensation of nuclei of neutrophils, strongly indicating the formation of NETs in lungs of both Wt and *mrp14^−/−^* mice (Fig. S3). It has recently been shown that MRP8/14 is abundantly present in NETs and that its presence is critical for fungal clearance [24], [27]. To test whether MRP8/14 is important in NET-mediated growth inhibition of *K. pneumoniae*, we stimulated Wt and *mrp14^−/−^* neutrophils with PMA to form NETs and then incubated these with viable bacteria. NETs from Wt neutrophils inhibited the growth of *Klebsiella*, while *mrp14^−/−^* NETs did not (Fig. 9A). To investigate the antibacterial properties of MRP8/14 in human NETs, we induced NET formation in neutrophils from healthy donors and then coincubated these with viable *Klebsiella* in the presence or absence of a neutralizing polyclonal anti-MRP14 antibody. In line with results obtained with other pathogens [24]–[27], human NETs effectively inhibited the growth of *Klebsiella*. This effect was strongly dependent on MRP8/14: addition of an anti-MRP14 antibody, blocking the chelating effect of MRP8/14 [27], almost completely restored growth of *K. pneumoniae*. These data confirm that NET-mediated growth inhibition of *K. pneumoniae* in a human system is MRP8/14 dependent. This growth inhibition may rely on the chelation of divalent cations, since addition of zinc in excess led to the same, if not an even stronger effect on growth of *Klebsiella* (Fig. 9B). The growth-inhibiting role of MRP8/14 within NETs also applied to other bacteria that are sensitive to NETs. Both gram-positve *S. aureus* and gram-negative *Pseudomonas aeruginosa*, showed an increased outgrowth in the presence of anti-MRP14 compared to control IgG (Fig. S4).

## Discussion

Gram-negative sepsis is a major challenge in the care of critically ill patients. Despite the availability of effective antimicrobial therapy and supportive care, mortality rates remain up to 30–50% [3], [41]. Severe sepsis is associated with the release of MRP8/14 [16], which in models of endotoxic shock and fulminant sepsis contributes to organ injury and mortality [15], [16]. The clinical scenario of sepsis, however, involves an initially localized infectious source with subsequent spreading of bacteria to distant body sites. We argued that in this setting, quite different from its previously described detrimental role in acute systemic sepsis models, MRP8/14 may be an important component of protective innate immunity at least in part because of its antimicrobial properties [21]–[23]. Thus, in the present study we aimed to determine the role of MRP8/14 during gram-negative sepsis originating from the lungs, using an established clinically relevant pneumonia model characterized by gradual growth of bacteria at the primary site of infection followed by dissemination, tissue injury and death [36]–[38], allowing to study a potential role of MRP8/14 in both the initial immune response as well as the subsequent harmful systemic inflammation phase. In summary, we found that intranasal *K. pneumoniae* infection resulted in local and systemic MRP8/14 release and that MRP14 deficiency led to an increased mortality, most likely as a consequence of enhanced bacterial outgrowth and organ damage. The beneficial role of this heterodimer may result from its involvement in phagocytosis and its strong growth inhibitory effect, while it hardly influenced the TLR4 mediated cytokine response to *Klebsiella*.

Severe human sepsis results in systemic release of MRP8/14 irrespective of the primary source of infection [16]. Patients with sepsis caused by pneumonia display the highest protein levels [16]. In the present mouse study, systemic MRP8/14 levels gradually increased during the course of the infection. Our current finding of high MRP8/14 concentrations at the primary site of infection from 24 hours onward is in accordance with a previous investigation from our group reporting high local levels of MRP8/14 in patients and mice with bacterial peritonitis [16]. Of note, however, systemic MRP8/14 levels in mice with *E. coli* peritonitis were at least ten times higher after one day than in mice with *Klebsiella* pneumonia studied here, which most likely reflects the fulminant nature of the septic syndrome induced by intraperitoneal *E. coli* administration. In these acute challenge models, such as is also produced by high dose LPS injection, MRP8/14 was suggested to act as a danger signal enhancing the cytokine response of LPS via TLR4, thereby potentiating the harmful systemic inflammatory response syndrome [15], [16]. Indeed, after either *E. coli* or LPS administration, MRP14 deficiency attenuated systemic inflammation and consequently improved survival in *E. coli* induced peritonitis and LPS-induced shock, starting to occur from 20 and 6 hours respectively [15]. In contrast, in the more clinically relevant sepsis model used here, a “cytokine storm” such as detected after LPS administration was not induced and mortality only started to occur after 2 days, allowing the gradually increasing levels of MRP8/14 to serve its beneficial role in antimicrobial defense.

Of note, whereas MRP14 deficiency clearly reduced systemic cytokine release after LPS or *E. coli* injection [15], [16], such an effect was not seen during *Klebsiella* pneumonia. In contrast, plasma concentrations of proinflammatory cytokines were higher in *mrp14^−/−^* mice during late stage infection, whereas lung cytokine and chemokine levels were largely similar in *mrp14^−/−^* and Wt mice. The increased plasma levels of IL-1β and IL-6 in *mrp14^−/−^* mice likely reflect the increased bacterial loads in blood, providing a more potent proinflammatory stimulus. In accordance with our *in vivo* data, MRP14 deficiency had little or no effect on the TLR4 mediated TNF-α response in a 6 hour incubation of whole blood with growth-arrested *K. pneumoniae*. Together, these data suggest that MRP8/14 minimally contributes to the cytokine response in the context of a gradually growing bacterial load, *i.e.* can be compensated for by other mechanisms. Furthermore, lungs from *mrp14^−/−^* mice showed increased inflammation, most likely as a consequence of the more severe infection in these animals and indicating that MRP8/14 is not a critical inducer of lung inflammation during gram-negative pneumosepsis.

In spite of high lung concentrations of MRP8/14, this heterodimer did not play a significant role in the control of local infection considering that pulmonary bacterial loads only slightly differed between *mrp14^−/−^* and Wt mice after 24 and 48 hours. The lack of MRP8/14 did however result in strongly increased bacterial burdens in blood, spleen and especially the liver. This led us to postulate that the apparent antimicrobial effect of MRP8/14 primarily resided in organs distant from the lung. Indeed, *mrp14^−/−^* mice administered with *K. pneumoniae* directly intravenously, thereby bypassing a potential effect of MRP8/14 in the airways, displayed strongly increased bacterial growth in multiple body sites, including the spleen and liver. We found a reduced capacity of *mrp14^−/−^* macrophages, but not *mrp14^−/−^* neutrophils, to phagocytose *Klebsiella in vitro*, which may partially explain enhanced bacterial outgrowth in these organs. Indeed, resident spleen macrophages form an important barrier to blood-borne pathogens and facilitate clearance in systemic infection [42]. The vast majority of bacteria entering the bloodstream are cleared by the liver [43]. Resident liver macrophages (Kupffer cells), constitute 80–90 percent of total tissue macrophages in the body [44] and have been attributed to clear the bulk of bacteria that are taken up by this organ [43]. A number of more recent studies, however, show that certain pathogens, including *K. pneumoniae*, can be cleared even in animals that lack Kupffer cells and suggest that immigrating neutrophils crucially contribute in hepatic clearance of circulating bacteria [45]–[47]. Hence, the exact contribution of MRP14 mediated phagocytosis by macrophages in our *in vivo* model remains to be elucidated in further detail.

Impaired antibacterial defense in the liver may have led to enhanced formation of (micro)abscesses. Abscesses, contain high levels of MRP8/14 and could be essential in the control of *K. pneumoniae* infection [22], [48]. We therefore hypothesize that liver abscesses deficient for MRP8/14 promote bacterial outgrowth of *Klebsiella* and may be the source for further bacterial dissemination. As a consequence of uncontrolled liver infection, enhanced hepatocellular damage occurred as reflected by the increased plasma levels of liver transaminases and enhanced liver pathology.

Several animal studies have implicated MRP8/14 as a mediator of neutrophil recruitment. In murine air pouch models, pretreatment with blocking antibodies directed against MRP8 and MRP14 significantly reduced leukocyte migration in response to LPS [17] or monosodium urate crystals [18]. Anti-MRP8 and anti-MRP14 antibodies also attenuated leukocyte influx into the pulmonary compartment during *S. pneumoniae* pneumonia [20]. Chemotaxis by MRP8/14 may partly act via upregulation of adhesion molecule expression and induction of CXC chemokines [35]. In the present study we did not find evidence for a role for MRP8/14 in chemotaxis: MPO levels in whole lung homogenates and the number of Ly-6G positive cells in lung tissue slides were not affected by the loss of MRP8/14 in spite of reduced levels of the neutrophil attracting chemokine MIP-2 early after infection. Similarly, we showed earlier that neutrophil numbers in the peritoneal cavity during *E. coli* induced peritonitis did not differ between *mrp14^−/−^* and WT mice [16]. MRPs may also mediate other neutrophil functions like, degranulation phagocytosis and respiratory burst [39], [49]. We however, found a similar capacity of *mrp14^−/−^* and Wt neutrophils to mount a respiratory burst (data not shown) and to phagocytose when incubated with *Klebsiella* bacteria.

Recently, MRP8/14 was found to inhibit *Staphylococcus (S.) aureus* growth through chelation of zinc and manganese [22]. Divalent ion-chelation also reduced the enzymatic activity of superoxide dismutase thereby inhibiting bacterial virulence [23]. In accordance, *mrp14^−/−^* mice showed higher bacterial loads after intravenous *S. aureus* injection [22]. In contrast, mouse studies investigating abdominal sepsis or urinary tract infection caused by *E. coli* [16], [50] or pneumonia caused by *S. pneumoniae* [20] did not point to an antimicrobial role for MRP8/14. In the present study we showed that the growth of *K. pneumoniae* was dose dependently inhibited by MRP8/14 while neither MRP8 nor MRP14 homodimers affected growth in any way. The antimicrobial effect of MRP8/14 toward *Klebsiella* could be overcome by addition of zinc implicating chelation of divalent cations by MRP8/14 as a key process herein. During infection, neutrophils can kill pathogens through different mechanisms, including by the release of NETs composed of chromatin decorated with neutrophil derived proteins [24]–[26]. We here observed the presence of decondensated nuclei of neutrophils in the lungs strongly indicating the formation of NETs. Histones have been implicated as the predominant antibacterial component of NETs, responsible for a reduction in bacterial counts *in vitro* already after 30 minutes [25]. Such a role in a short time span was not found for MRP8/14: incubation of *Klebsiella* with NETs for one hour resulted in reduced outgrowth as well, but this was not influenced by coincubation with anti-MRP14 (data not shown). A previous investigation established that MRP14 is not required for NET formation by neutrophils [24]. We here show that MRP8/14 is an important player in growth inhibition in late stage infection of mouse NETs and that NETs void of MRP8/14 are unable to inhibit the growth of *K. pneumoniae*. NETs in the *Klebsiella*-infected lungs, which have been documented in a previous study [40], might contribute to decreased dissemination into spleen and liver in an MRP8/14-dependent manner via this growth inhibitory effect. In accordance, human NETs strongly inhibited *Klebsiella* growth, which was almost completely reversed by anti-MRP14 antibodies blocking the chelating effect of MRP8/14 or by the addition of zinc. Our current data are the first to indicate that the capacity of NETs to kill a bacterium is highly dependent on MRP8/14 and that MRP8/14 exerts its antimicrobial effects in NETs on *Klebsiella* through metal chelation. A similar MRP8/14 dependent mechanism was recently shown for killing by NETs of the fungi *Candida albicans* [24] and *Aspergillus nidulans* [27].

In conclusion, we here document that MRP14 deficiency leads to increased bacterial growth and dissemination accompanied by enhanced organ damage and mortality in *K. pneumoniae* sepsis originating from the lungs. MRP8/14 exerts its essential protective role by its involvement in macrophage phagocytosis and by directly inhibiting the growth of *K. pneumoniae* through divalent cation chelation. This study shows for the first time that MRP8/14 within NETs is critical in both a murine and human system controlling bacterial infection.

## Materials and Methods

### Ethics statement

Experiments were carried out in accordance with the Dutch Experiment on Animals Act and approved by the Animal Care and Use Committee of the University of Amsterdam (Permit number: DIX100121, DIX101223) or carried out according to the recommendations in the guide for the care and use of laboratory animals conformed to Swedish animal protection laws and applicable guidelines (djurskyddsmyndigheten DFS 2004:4) and approved by the local Ethical Committee (Dnr A 29-09).

### Mice

C57Bl/6 Wild type (Wt) mice were purchased from Charles River Laboratories Inc. (Maastricht, the Netherlands). *Mrp14^−/−^* mice, backcrossed >10 times to a C57BL/6 background were generated as described [28] and bred in the animal facility of the Academic Medical Center (Amsterdam, the Netherlands). The Animal Care and Use Committee of the University of Amsterdam approved all experiments.

### Design

Mice were intranasally inoculated with 10^4^ *K. pneumoniae* serotype 2 (ATCC 43816 Rockville, MD) in a 50 µl saline solution (n = 7–8 per strain) and sacrificed 6, 24 or 48 hours thereafter [36], [37]. In an additional study, mice were intravenously injected with *K. pneumoniae* (2×10^3^ colony forming units (cfu)) in the tail vein and sacrificed 48 hours after infection. Collection and handling of samples were done as previously described [36], [37]. In brief, blood was drawn into heparinized tubes and organs were removed aseptically and homogenised in 4 volumes of sterile isotonic saline using a tissue homogenizer (Biospec Products, Bartlesville, UK). To determine bacterial loads, ten-fold dilutions were plated on blood agar (BA) plates and incubated at 37°C for 16 h. In survival studies mice (n = 12 to 16 per strain) were intranasally inoculated with 10^3^ or 10^4^ *K. pneumoniae* and monitored for up to 10 days after infection. Bronchoalveolar lavage (BAL) fluid was obtained from a separate group of infected Wt mice (n = 6) at indicated time points. The trachea was exposed through a midline incision and cannulated with a sterile 22-gauge Abbocath-T catheter (Abbott Laboratories, Sligo, Ireland). Bilateral BAL was performed by instilling two 0.5 ml aliquots of sterile phosphate buffered saline (PBS) as described earlier [36]. 0.9–1 ml of BAL fluid was retrieved per mouse.

### Assays

Lung homogenates were prepared for immune-assays as described before [36], [37]. MRP8/14 levels were measured by ELISA [15]. Lung cytokines and chemokines TNF-α, interleukin (IL)-1-β, IL-6, IL-10, Keratinocyte-derived chemokine (KC) and macrophage inflammatory protein 2 (MIP-2)(all R&D systems, Minneapolis, MN) and Myeloperoxidase (MPO; Hycult Biotechnology BV, Uden, the Netherlands) were measured using specific ELISAs according to manufacturer's recommendations. Plasma TNF-α, IL-6 and IL-1β were measured by cytometric bead array flex set assay (BD Biosciences, San Jose, CA) in accordance to the manufacturer's instructions. Lactate dehydrogenase (LDH), aspartate aminotransferase (AST) and alanine transaminase (ALT) were measured in plasma with kits from Sigma (St. Louis, MO), using a Hittachi analyzer (Boehringer Mannheim, Mannheim, Germany).

### Histology

Lung and liver pathology scores were determined as described before [36]–[38]. In brief, lungs and livers were harvested at the indicated time points, fixed in 10% buffered formalin, and embedded in paraffin. 4 µm sections were stained with haematoxylin and eosin (HE) and analyzed by a pathologist blinded for groups as described earlier. To score lung inflammation and damage, the entire lung surface was analyzed with respect to the following parameters: bronchitis, edema, interstitial inflammation, intra-alveolar inflammation, pleuritis, endothelialitis and percentage of the lung surface demonstrating confluent inflammatory infiltrate. Each parameter was graded 0–4, with 0 being ‘absent’ and 4 being ‘severe’. Livers were scored according to the following parameters: number of thrombi, number of (micro)abscesses, presence and degree of inflammation, and presence and degree of necrosis. Each parameter was graded 0–3, with 0 being absent and 3 being severe. The total pathology score for lungs and livers was expressed as the sum of the score for all parameters. Granulocyte staining was done using FITC-labeled rat anti-mouse Ly-6 mAb (Pharmingen, San Diego, CA) as described earlier [51]. Ly-6G expression in the lung tissue sections was quantified by digital image analysis [52]. In short, lung sections were scanned using the Olympus Slide system (Olympus, Tokyo, Japan) and TIF images, spanning the full tissue section were generated. In these images Ly-6G positivity and total surface area were measured using Image J (U.S. National Institutes of Health, Bethesda, MD, http://rsb.info.nih.gov/ij); the amount of Ly-6G positivity was expressed as a percentage of the total surface area. MRP8 and MRP14 staining of lung tissue were performed as described previously [28]. For immunostainings, specimens were processed similarly as described previously [24]. Briefly, samples were deparaffinized, rehydrated in decreasing concentrations of EtOH, and subjected to antigen retrieval by cooking in 10 mM citrate buffer, pH 6.0, for 10 min. Specimens were blocked with 2% BSA and mouse Ig blocking reagent according to manufacturer's protocol (Vector Laboratories, Burlingame, USA) in PBS/0.1% Triton for 1 h at room temperature. Subsequently, specimens were incubated with primary antibodies directed against myeloperoxidase (MPO) (A0398, Dako) and histone H1 (clone AE-4, Acris) diluted in blocking solution over night at 4°C. Primary antibodies were detected with Alexa Fluor 488- and 568-conjugated secondary antibodies (Life Technologies) diluted in 2% BSA in PBS/0.1% Triton. DNA was visualized with DAPI (Life Technologies) and slides were mounted with fluorescence mounting medium (Dako). Pictures were taken with a Nikon C1 confocal microscope and presented as maximum intensity projections from parts of Z-stacks.

### Whole blood stimulation

Growth-arrested bacteria were prepared as described [53] In brief, *K. pneumoniae* were cultured and washed with pyrogen-free sterile saline and resuspended in sterile PBS to a concentration of 2×10^9^ bacteria/ml. The concentrated *K. pneumoniae* preparation was treated for 1 h at 37°C with 50 µg/ml Mitomycin C (Sigma-Aldrich; Zwijndrecht, the Netherlands) to prepare alive but growth-arrested bacteria. Subsequently, the growth-arrested *K. pneumoniae* preparation was washed twice in ice-cold sterile PBS by centrifugation at 4°C, and the final pellet was dispersed in ice-cold PBS in the initial volume and transferred to sterile tubes. Undiluted samples of these preparations failed to generate any bacterial colonies when plated on BA plates, indicating successful growth arrest. Bacteria were washed and resuspended in RPMI and diluted to ten-fold lower bacterial concentrations (2×10^4–7^ cfu/ml). 100 µl of heparinized whole blood obtained from 4 individual Wt, *mrp14^−/−^* and *tlr4^−/−^* [54] mice were then incubated with 100 µl of the different bacterial concentrations in a 96 wells plate and incubated for 6 hours at 37°C, 5% CO2. After incubation, plates were centrifuged at 4°C and supernatant was harvested for determination of TNF-α.

### Phagocytosis assay

Phagocytosis of *K. pneumoniae* was determined as described before [55]. In brief, growth-arrested bacteria were prepared as described above and labeled with carboxyfluorescein succinimidyl ester (CFSE, Invitrogen, Breda, the Netherlands). 50 µl heparinized whole blood was incubated with 50 µl bacteria in IMDM (Gibco) (end concentration of 1×10^7^ CFU/ml) at 37°C (n = 6 per group) or 4°C (n = 3 per group). After 20 minutes, samples were put on ice to stop phagocytosis. Afterwards, red blood cells were lysed using isotonic NH_4_Cl solution (155 mM NH_4_Cl, 10 mM KHCO_3_, 100 mM EDTA, pH 7.4). Neutrophils were labeled using anti-Gr-1-PE (BD Pharmingen, San Diego, CA) and washed twice in FACS-buffer (0.5% BSA, 0.01% NaN3, 0.35 mM EDTA in PBS) for analysis. Peritoneal macrophages (derived from 3 mice) were pooled, washed twice and resuspended in IMDM containing 2 mM L-glutamine and 10% fetal calf serum (FCS) (Gibco). 1×10^5^ cells per well were seeded in a 96-well flat-bottom plate in 250 µL to adhere overnight at 37°C, 5% CO_2_. The following day, macrophages were washed twice with pre-warmed medium to wash away non-adherent cells. Growth-arrested bacteria were opsonised in 10% normal mouse serum before added to cells at a multiplicity of infection of 100 in a volume of 100 µL. Bacteria and macrophages were spun at 1000 RPM for 5 minutes and incubated at 37°C (n = 8 wells per strain) or 4°C (n = 4 wells per strain). After 1 hour, samples were washed twice with ice-cold PBS, then thoroughly scraped from the bottom and washed again in FACS-buffer. The degree of phagocytosis was determined using FACSCalibur (Becton Dickinson Immunocytometry, San Jose, CA.) The phagocytosis index of each sample was calculated as follows: geo mean fluorescense×% positive cells.

### Growth inhibition by MRPs

Recombinant mouse MRP8 and MRP14 homodimers as well as MRP8/14 heterodimers were generated as previously described [56]. To test growth inhibitory effects of MRPs on *K. pneumoniae*, bacteria were grown to log phase and diluted to approximately 10.000 cfu/ml in HEPES buffered RPMI. 100 µl of this bacterial suspension was added to 100 µl of recombinant murine MRP8 or 14 homodimer or MRP8/14 heterodimer in HBSS (end concentration 50 µg/ml unless indicated otherwise; n = 4–6) without Ca^2+^ and Mg^2+^ (Gibco). Bacteria and MRPs were incubated for 0, 2, 4, 8 or 24 hours at 37°C. To test reversibility of growth inhibitory effects on *K. pneumoniae*, increasing concentrations of ZnSO_4_ were added to a 10 µg/ml MRP8/14 solution. Growth was assessed by plating out ten-fold dilutions of bacterial concentrations on BA plates and incubation at 37°C for 16 h.

### Growth inhibition by murine NETs

Murine neutrophils were isolated from Wt and *mrp14^−/−^* mice; animals used for these experiments were bred in the animal facility of the Umeå University (Umeå, Sweden). Experiments were carried out according to the recommendations in the guide for the care and use of laboratory animals conformed to Swedish animal protection laws and applicable guidelines (djurskyddsmyndigheten DFS 2004:4) and were approved by the local ethics committee (Dnr A 29-09). Mature murine neutrophils were isolated from bone marrow as previously described [57]. Briefly, bone marrow cells from tibia and femur were singularized by using a 70-µm cell strainer and separated by centrifugation for 30 minutes at 1500 *g* on a discontinuous Percoll gradient with 52% (vol/vol), 69% (vol/vol), and 78% (vol/vol). Neutrophils harvested from the distinct layer between 69% and 78% were resuspended in HBSS without Ca^2+^ and Mg^2+^ until use. Murine NETs were induced as described earlier [24], [27], [57]. In a 24-well plate, 5×10^5^ Wt or *mrp14^−/−^* mouse neutrophils in 500 µL RPMI with 1% (vol/vol) mouse serum were stimulated with 100 nM phorbol 12-myristate 13-acetate (PMA, Sigma Aldrich, St. Louis, MO) for 20 hours at 37°C, 5% CO_2_ to induce NET formation. The supernatant was discarded; NETs were washed once with RPMI and incubated with 500 µL RPMI containing approximately 5000 cfu *K. pneumoniae*. Subsequently plates were centrifuged for 5 minutes at 300 *g* and incubated for 7 hours at 37°C.

### Growth inhibition by human NETs

Human neutrophils were isolated from peripheral blood of healthy donors using Polymorphprep (Axis-Shield, Oslo, Norway) according to the manufacturer's instructions. Neutrophils were harvested and washed with HBSS without Ca^2+^ and Mg^2+^. Remaining red blood cells were lysed using sterile isotonic NH_4_Cl solution without EDTA for 10 minutes. After lysis of red blood cells, neutrophils were washed and resuspended in HBSS without Ca^2+^ and Mg^2+^ until use. In a 96-well plate, 3×10^5^ human neutrophils resuspended in 50 µL HEPES buffered RPMI (Gibco) were stimulated with 100 nM PMA for 4 hours at 37°C, 5% CO_2_ to induce NET formation [25]. Sytox Green (Molecular Probes, Carlsbad, CA) confirmed the presence of NETs (data not shown). The supernatant was discarded and approximately 100 cfu log phase grown *K. pneumoniae*, *P. aeruginosa* or *S. aureus*, resuspended in 200 µL HEPES buffered RPMI were added to the wells and spun down for 5 minutes at 300 *g*. To test growth inhibiting properties of endogenous MRP8/14 in human NETs, samples were pre-incubated for 30 minutes with 15 µg/ml rabbit polyclonal anti-MRP14 antibody (H00006280-D01P; Abnova, Taipei, Taiwan), unspecific rabbit polyclonal control antibody or an excess of ZnSO_4_ (1 mM), before the addition of bacteria. Overnight bacterial growth was assessed by plating out ten-fold dilutions of bacterial concentrations on BA plates and incubation at 37°C for 16 hours. Bacterial growth of *Klebsiella* is expressed as percentage of control values (*K. pneumoniae* growth in media without neutrophils with or without anti-MRP14, control antibody or ZnSO_4_).

### Statistical analysis

Data are expressed as box-and-whisker diagrams depicting the smallest observation, lower quartile, median, upper quartile and largest observation unless indicated otherwise. Differences between *mrp14^−/−^* and Wt mice were analyzed by Mann-Whitney U test. Survival was compared by Kaplan-Meier analysis followed by a log rank test. Analyses were done using GraphPad Prism version 5.0, Graphpad Software (San Diego, CA). Values of p<0.05 were considered statistically significant different.

## Supporting Information

Figure S1 ***Mrp14^−/−^*** **mice show enhanced bacterial dissemination after intravenous** ***K. pneumoniae*** **infection.** Bacterial loads in blood (A), lung (B), spleen (C) and liver (D) of *K. pneumoniae* in Wt (grey) and *mrp14^−/−^* mice (white) 48 hours after infection. Data are expressed as box-and-whisker diagrams depicting the smallest observation, lower quartile, median, upper quartile and largest observation (8 mice per group). **p<0.01, ***p<0.001 versus Wt mice
(TIF)

Figure S2 **MRP14 deficiency does not reduce cytokine response in whole blood to** ***Klebsiella*** **infection.** TNF-α levels after a 6 hour stimulation of whole blood obtained from individual Wt, *mrp14^−/−^* and *tlr4^−/−^* mice (n = 4 per group) with log increasing concentrations of growth-arrested *K. pneumoniae*.
(TIF)

Figure S3 **Decondensed nuclei of neutrophils in lungs of Wt and m** ***rp14^−/−^*** **mice are indicators for the release of NETs.** Representative confocal immunofluorescence images of lung sections of Wt (A–D) and m*rp14^−/−^* mice (E–H) 24 hours after *K. pneumoniae* infection stained with DAPI (blue) and primary antibodies against MPO (red) and histone H1 (green). In both Wt and m*rp14^−/−^* lungs we found similar amounts of decondensed nuclei from neutrophils (arrows), a prior stage of NET formation. Scale bars indicate 10 µm.
(TIF)

Figure S4 **Bacterial growth inhibition by human NETs is MRP8/14 dependent.** 5×10^5^ human neutrophils were induced to make NETs and pretreated with a rabbit polyclonal anti-MRP14 antibody (α-MRP14) or an unspecific rabbit polyclonal control antibody (control IgG) and then infected with 100 cfu *Staphylococcus aureus* (A) or *Pseudomonas aeruginosa* (B). Cfu counts were determined after incubation of 15 hours (B). Data are expressed as box-and-whisker diagrams depicting the smallest observation, lower quartile, median, upper quartile and largest observation of at least 5 replicates. *p<0.05 versus controls.
(TIF)
