# Supplementary figures and images for: Myeloid-Related Protein-14 Contributes to Protective Immunity in Gram-Negative Pneumonia Derived Sepsis
Source: PLoS Pathog. 2012 Oct 25;8(10):e1002987. doi: 10.1371/journal.ppat.1002987 (PMC3486918; doi:10.1371/journal.ppat.1002987)

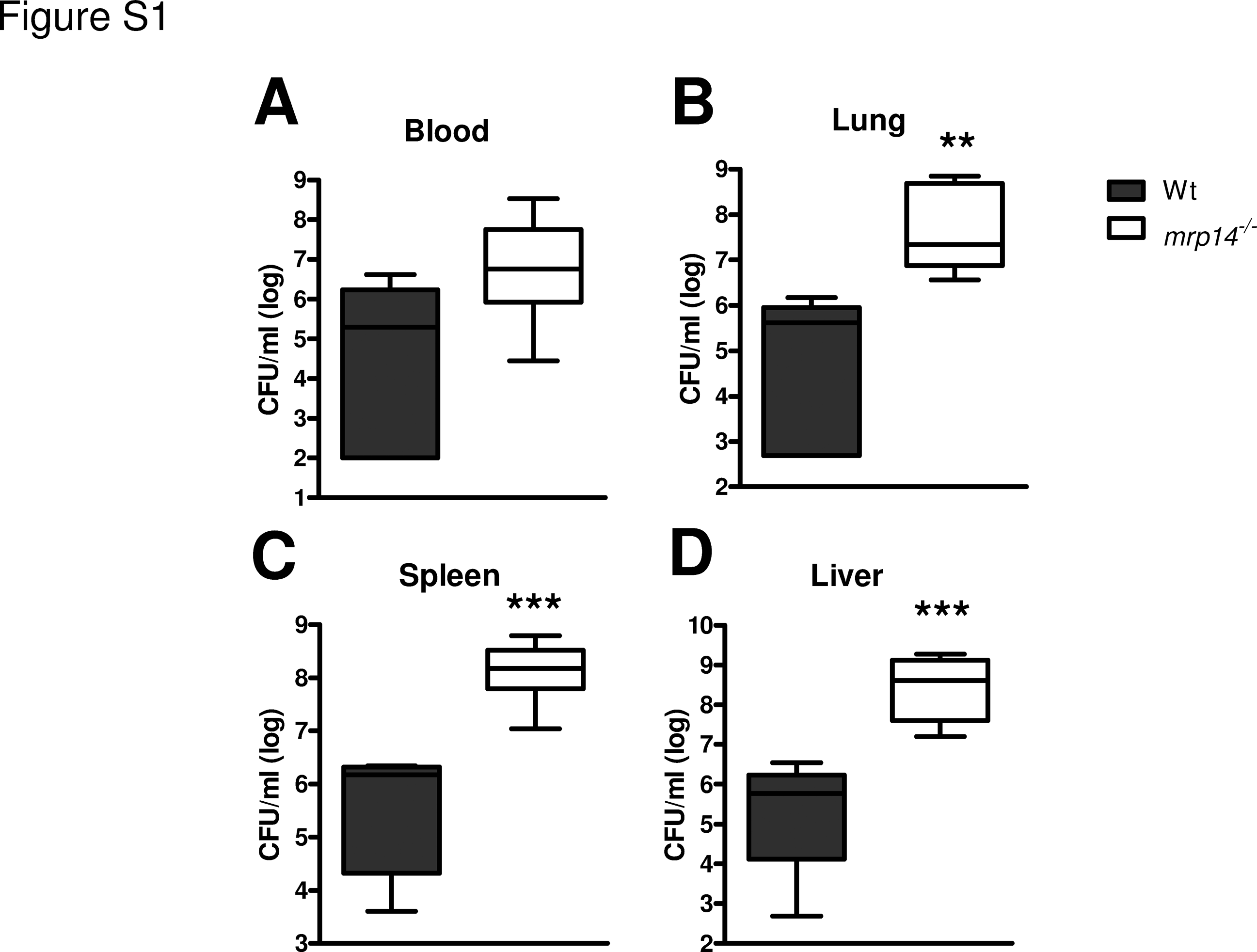

Supplement: Figure S1 — Mrp14−/− mice show enhanced bacterial dissemination after intravenous K. pneumoniae infection. Bacterial loads in blood (A), lung (B), spleen (C) and liver (D) of K. pneumoniae in Wt (grey) and mrp14−/− mice (white) 48 hours after infection. Data are expressed as box-and-whisker diagrams depicting the smallest observation, lower quartile, median, upper quartile and largest observation (8 mice per group). **p<0.01, ***p<0.001 versus Wt mice (TIF) [file ppat.1002987.s001.tif]

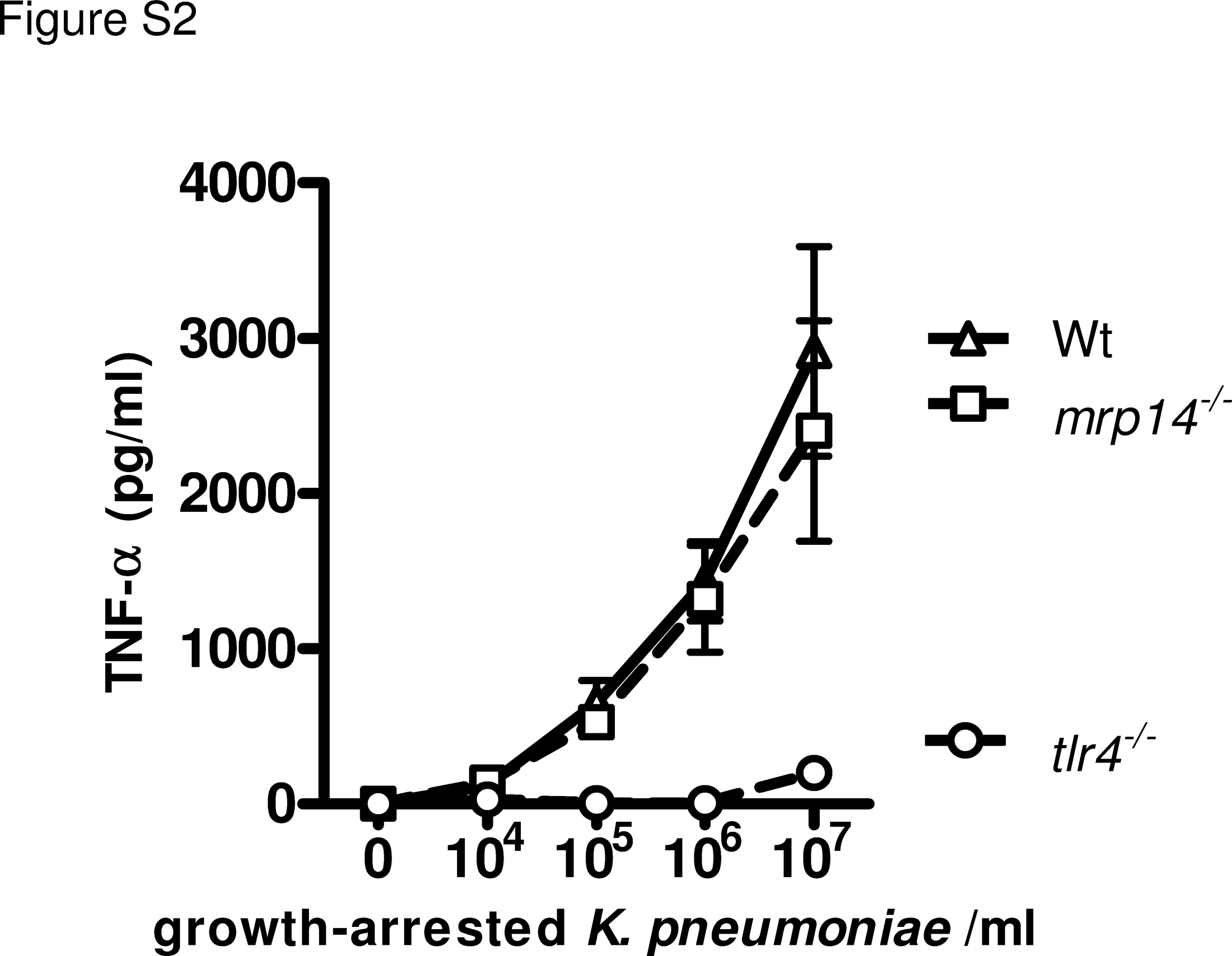

Supplement: Figure S2 — MRP14 deficiency does not reduce cytokine response in whole blood to Klebsiella infection. TNF-α levels after a 6 hour stimulation of whole blood obtained from individual Wt, mrp14−/− and tlr4−/− mice (n = 4 per group) with log increasing concentrations of growth-arrested K. pneumoniae. (TIF) [file ppat.1002987.s002.tif]

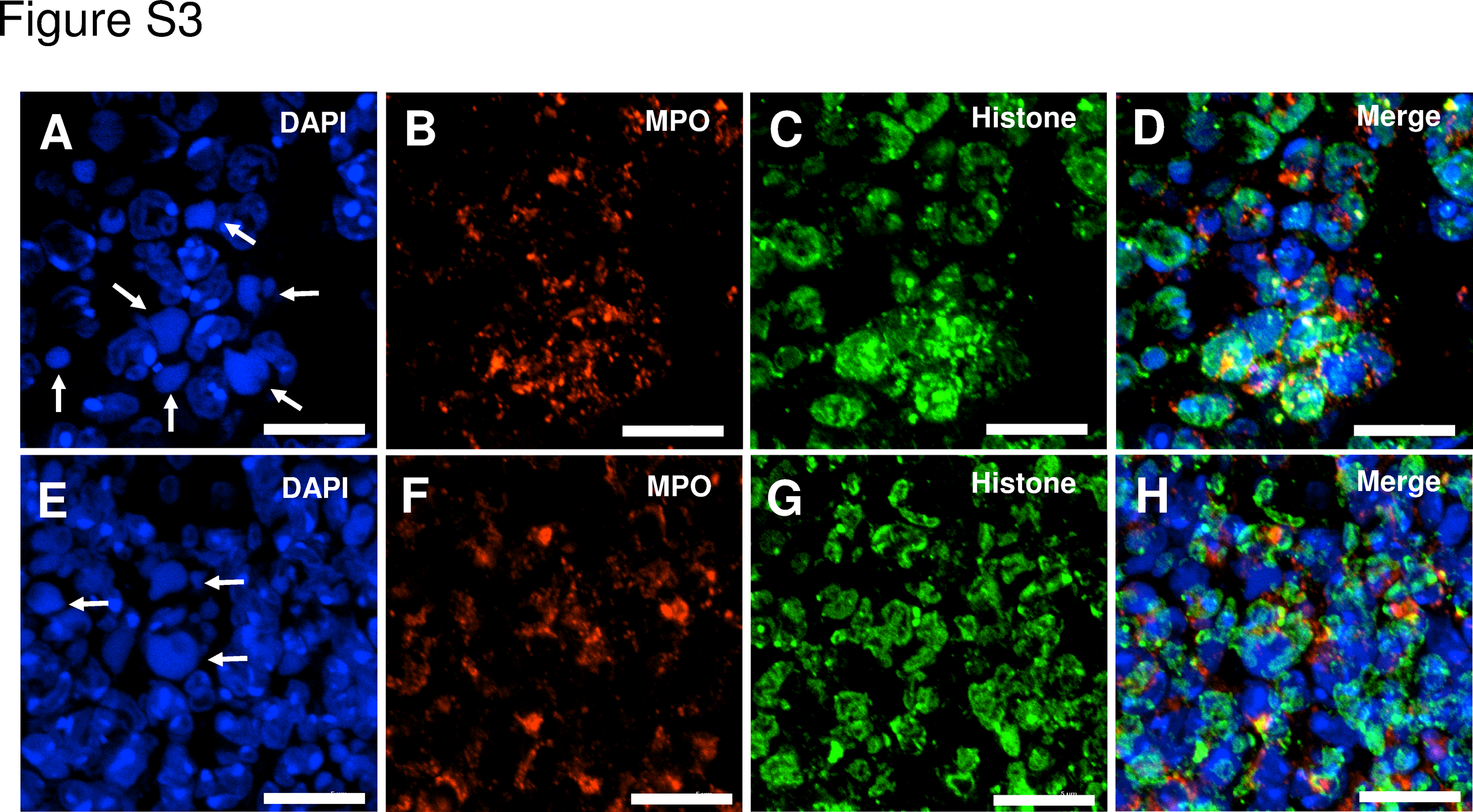

Supplement: Figure S3 — Decondensed nuclei of neutrophils in lungs of Wt and m rp14−/− mice are indicators for the release of NETs. Representative confocal immunofluorescence images of lung sections of Wt (A–D) and mrp14−/− mice (E–H) 24 hours after K. pneumoniae infection stained with DAPI (blue) and primary antibodies against MPO (red) and histone H1 (green). In both Wt and mrp14−/− lungs we found similar amounts of decondensed nuclei from neutrophils (arrows), a prior stage of NET formation. Scale bars indicate 10 µm. (TIF) [file ppat.1002987.s003.tif]

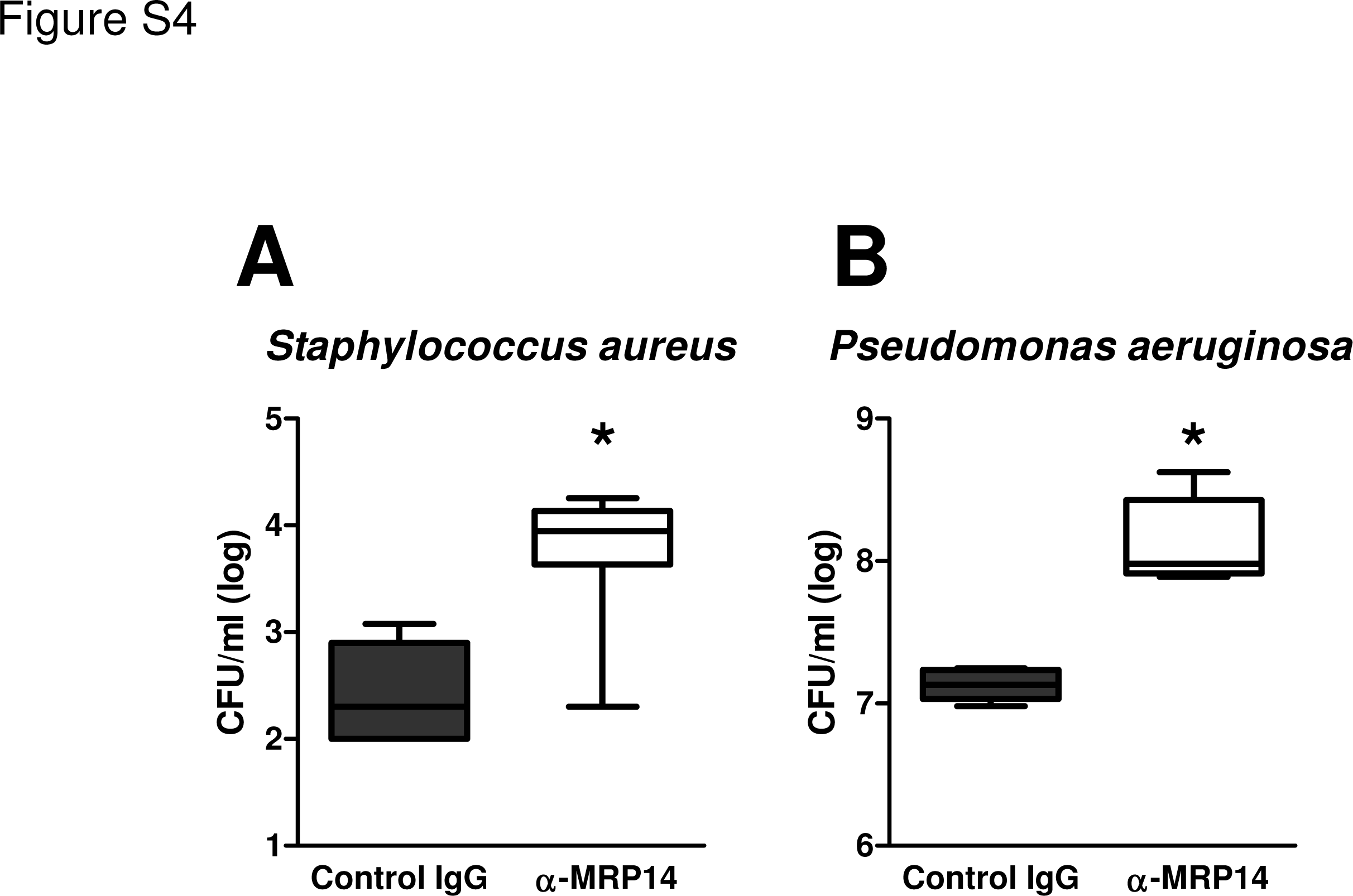

Supplement: Figure S4 — Bacterial growth inhibition by human NETs is MRP8/14 dependent. 5×10^5^ human neutrophils were induced to make NETs and pretreated with a rabbit polyclonal anti-MRP14 antibody (α-MRP14) or an unspecific rabbit polyclonal control antibody (control IgG) and then infected with 100 cfu Staphylococcus aureus (A) or Pseudomonas aeruginosa (B). Cfu counts were determined after incubation of 15 hours (B). Data are expressed as box-and-whisker diagrams depicting the smallest observation, lower quartile, median, upper quartile and largest observation of at least 5 replicates. *p<0.05 versus controls. (TIF) [file ppat.1002987.s004.tif]
